# Review of Socioeconomic Disparities in Lower Extremity Amputations: A Continuing Healthcare Problem in the United States

**DOI:** 10.7759/cureus.3418

**Published:** 2018-10-05

**Authors:** Raghavendra L Girijala, Ruth L Bush

**Affiliations:** 1 Medical Education and Simulation, Texas A&M University, College Station, USA; 2 Surgery, University of Houston College of Medicine, Houston, USA

**Keywords:** amputation, disparity, extremity, socioeconomic

## Abstract

Lower extremity amputation is one of the most unfortunate, yet preventable, consequences of uncontrolled lower limb ischemia occurring secondary to diabetes mellitus or peripheral arterial disease. In the United States, racial and socioeconomic disparities are associated with significant differences seen in the incidence and type or level of lower extremity amputation among patients. Due to shifting demographics and the uncertain state of healthcare coverage, lower extremity amputation rates are only projected to increase in the future. Given the potential societal and individual costs associated with the loss of a limb, this review seeks to summarize the recent findings on disparities in the identification, treatments offered, and outcomes of lower limb ischemia in order to elucidate potential interventions at the practitioner and policy levels.

## Introduction and background

In 2005, the United States had 1.6 million amputees, equating to one in every 190 persons. By 2050, this number is projected to increase to over 3.6 million with an estimated incidence rate of 185,000 patients per year [1-2]. Limb loss is most often secondary to peripheral arterial disease (54%), followed closely by trauma (45%). With respect to peripheral arterial disease, the majority of patients have the concomitant diagnosis of diabetes mellitus [1]. The management of lower limb ischemia varies based on the racial group though, with worse outcomes more likely in minority groups. Previous studies have demonstrated that within African American populations, regardless of geographic region, there is a higher incidence of amputations compared to white populations (5.0-6.5 per 10,000 individual versus 1.2-2.5 per 10,000) [3]. Furthermore, African American persons are not only more likely to undergo limb amputation but also the amputations are more significant, with a higher risk of above-the-knee amputations [4-5].

The most recent United States Census data notes that one in three individuals identify as African American, Hispanic, or Native American [6]. Over the next three decades, it estimates this number to rise to one in every two persons, which highlights the urgency to address this issue both for individual patients and from a societal standpoint. Furthermore, projections estimate that by 2030, the number of those with diabetes worldwide will rise from 366 million to 552 million [7]. Given the increased risk of amputation in diabetic patients, there is potential that these alarming statistics actually underestimate the challenge our healthcare community will face in the upcoming decades [7-8]. This review will summarize several of the latest primary studies on health disparities as they relate to lower extremity amputation, with an emphasis on how the current findings can be translated into practical changes for the individual practitioner and the healthcare system.

## Review

Individual and societal cost

While there is debate regarding the exact degree and magnitude of change in incidence rates with lower extremity amputation, it is evident that the overall associated healthcare costs are continuing to rise. From a financial perspective, when accounting for these values, the cost cannot be limited solely to the pre-, peri-, and post-operative windows. Rather, ongoing costs in the form of long-term rehabilitation, care, and management must be considered.

With respect to procedural costs, estimates vary primarily due to the differences in the underlying data set, patient population, the timeframe of analysis, and charge calculations. Therefore, several studies will be compared to discuss overarching trends rather than focus on exact costs. According to the 2007 Inter-Society Consensus for the Management of Peripheral Arterial Disease, the average cost of an amputation was estimated to be $40,000 [9]. However, this value is for a single year and without context in terms of the level of amputation, complications, and follow-up care. In addition, a cross-sectional study from the Department of Veterans Affairs data provided an approximation of both overall amputation-associated cost and temporal change, albeit only for diabetes-related lower extremity amputations. In the fiscal year 2004, the cost estimate was $50,351 per patient for a cohort of 3,381 patients, equaling an overall cost of $170,236,037 [10]. Over the next half decade, these values increased to $60,647 per patient, with a total cost of $206,380,331 for 3,403 patients (all values expressed in 2012 dollars) [10]. However, like other studies, overall costs may have been underestimated, given that only costs associated with hospitalization were included.

Given these limitations in cost calculations, MacKenzie et al. studied the two-year direct cost and lifetime costs associated with lower extremity amputation, regardless of etiology or underlying disease process. Following 545 patients at level-1 trauma centers, they assessed the following variables: initial hospitalization, readmissions, inpatient rehabilitation, outpatient physician visits, physical and occupational therapy, and durable medical equipment cost. Based on these parameters, they ascertained the two-year cost to be $91,106 for index amputation and $509,275 for lifetime costs [11]. While the exact cost varies based on individual study methodology and calculation, it is clear that these estimates underscore the premise that lower extremity amputation is an expensive procedure for patients and healthcare systems. Furthermore, these costs are likely to increase given the widely acknowledged increasing prevalence of co-existing diabetes and peripheral arterial disease [11].

The impetus to prevent future amputations is not strictly economical but is also based on the suffering of the individual patient and potential outcomes. Quality of life scores have been noted to significantly drop for amputees compared to non-amputees, with changes in employment status and the use of prosthetic devices correlating with more negative scores [12]. As would be expected, amputees have also been found to have higher rates of depression, anxiety, and negative body image [13]. Even more striking, mortality risk varies between amputees and the general population. Based on the Centers for Medicare and Medicaid data from 2000 to 2008, mortality rates at one year and three years were 48.3% and 70.9% for amputees, respectively [14]. Comparatively, the one-year and three-year mortality rates for non-amputees were 24.2% and 43.2%, respectively [14]. However, these outcome measures are not necessarily equal among amputees. The lower extremity assessment project (LEAP), a multicenter, prospective observational study that attempted to determine who should undergo amputations, determined that poverty, lack of private insurance, and non-white race were determined to be predictors of worse health status post-amputation [15].

Current evidence of health disparities

Racial and socioeconomic disparities exist with respect to the incidence, prevalence, and management of limb ischemia, resulting in a variation in outcomes among certain sub-populations. While a number of studies over the last few decades have described these differences, the methods of data collection, analysis, and patient population studied varied significantly from study to study. Therefore, we will attempt to highlight the latest national studies performed with a similar methodology, to update the understanding of current disparities.

Populations at risk for limb amputation have been well documented, with temporal data being the most suited to analyze changing trends in these groups. From 1988 to 1996, the Healthcare Cost and Utilization Projection analyzed nationwide data and determined that amputations secondary to dysvascular conditions were overall on the rise. Upon data stratification by age and racial group, they noted that African American patients had higher rates of amputation, especially in older individuals [16]. Specifically, African American persons over 85 years were 11.7 times as likely as those younger to undergo an amputation due to dysvascular causes [16].

Considering the Medicare population only, one recent study sought to determine how amputation rates have changed over time, as well as the risk factors underlying these outcomes [17]. From 1999 to 2006, there were 23,976 amputations in Medicare recipients with diabetes. Of these, 11,558 were in high-risk patients, compared to 12,418 in low-risk patients (high risk was defined as end-stage renal disease patients or patients with three or more risk factors, including stroke, congestive heart failure, myocardial infarction, and ischemic heart disease) [17]. Although the authors concluded that lower extremity amputation rates decreased from 1999 to 2006 (4.8 per 1000 to 4.4 per 1000 patients), the proportion of amputees that were high risk with diabetes increased from 33% to 50% over the same time interval. Furthermore, comparing amputees to non-amputees, the African American race was more common in amputees (25.1% versus 12.6%). In addition, the African American race was associated with a higher incidence of amputation in both the low- and high-risk groups [17].

Variations in management

Studies have documented differences in the prevalence of amputations by race. More specifically, observations have highlighted higher rates of conversion of an at-risk patient to an amputee when the patient is of African American race [18-19]. However, factors outside of race likely play a confounding role in this higher rate, especially when considering potential differences in management. For example, one study by Holman and colleagues analyzed inpatient Medicare data from 2003 to 2006. In patients who underwent a major lower extremity amputation, they concluded that even after controlling for baseline patient characteristics, African American patients were less likely to have had a previous hospital admission for a limb-related disease or revascularization procedure before amputation than Caucasian patients [18]. Another study by Baser analyzed Medicare data from 2007 to 2008, noting that 0.41% of African Americans had a severe peripheral arterial disease or critical limb ischemia, double that of the Caucasian population (0.18%). More importantly, they noted that that African American patients had lower rates of revascularization and higher rates of amputation than Caucasian patients. This conclusion was also supported by the longer time to first revascularization and the shorter time to amputation in the African American cohort. Of note, these relationships remained statistically significant even after adjustment for age, gender, ischemic severity and other comorbidities [19].

The existence of racial disparity in amputations is further supported by a study from Hughes et al. that looked at the association of critical limb ischemia, amputation, and race. This study utilized the National Inpatient Sample (NIS) data from 1998-2005, which includes all-payer inpatient hospital stays data in the United States. Patients under 65 years of age and uninsured patients are included in this database. During the study period, 111,548 patients underwent amputations: 61% were Caucasians versus 25.4% African American. However, of the 240,139 admitted patients with the diagnosis of limb ischemia, 68.2% were Caucasian versus 19.5% African Americans [20]. Thus, although less than one in five admitted patients for limb ischemia is African American, over one in four undergoing amputation are African American. One potential explanation is that this patient population has lower rates of insurance (private or government) coverage compared to Caucasians [21]. Therefore, there may be a greater risk of patients losing continuous access to physicians and medications, potentially leading to greater disease severity on presentation.

With regards to whether management strategies differ by races, the results are highly controversial. There has been debate regarding the regional intensity of vascular care and its effect on amputation rates. Goodney et al. investigated Medicare patients who underwent peripheral vascular procedures prior to amputation from 2003 to 2006 and compared to future amputation rates from 2007 to 2009. When stratifying by geographical location, they saw large differences in amputation rates, ranging from one in 10,000 to 27 in 10,000 persons. In regions with high rates, African Americans comprised a larger proportion of the amputees. Furthermore, when the dataset was analyzed against the rates of vascular care, high rates of care were negatively correlated with amputation rates, specifically for outpatient diagnostic and therapeutic procedures and inpatient surgical revascularization. As a result, it logically follows that patients in regions with more vascular care were more likely to be offered revascularization, or limb salvage, prior to amputation [22]. These findings imply the potential benefits of targeting preventative care efforts to those regions with lower levels of vascular care and higher primary amputation rates.

Current climate and future interventions

Based on estimates from a decade-long (2001-2010) analysis of inpatient discharge records, major amputations have increased by 7.7% [23]. This analysis demonstrated that these admissions are more costly than ever, with values over $100,000 per patient [23]. Furthermore, despite technological advances, the rate of preventable amputations are not decreasing and patients with peripheral arterial disease and diabetes only continue to increase as our population ages [23-24].

Clearly, there is evidence, both historical and modern, that race plays a significant role in the incidence of lower extremity amputation. Data as recent as 2011 continues to demonstrate that African Americans have a higher risk of amputations, with a variety of contributing factors, including the condition of patients on admission [25-27]. Furthermore, given the impact of socioeconomic status on amputation risk, proposed interventions must keep these influences in mind in order to be effective [18-19]. A recent British study by Amin et al. confirmed this association, concluding that lower socioeconomic status (based on median household income in their neighborhood of residence) correlated with higher rates of lower extremity amputation, with the lowest quintile having an 8% greater incidence rate than the highest quintile (27.0 vs. 19.3 per 10,000 patients) [28]. Interestingly, this relationship remained constant even after adjusting for the utilization of primary care and variables such as differences in health literacy and ability to consistently afford medication and care [28].

Many interventions have previously been proposed regarding what physicians and other healthcare staff can do to prevent lower extremity amputation. While these studies are effective in proposing modifications to physician behaviors to improve the identification and prevention of complications that can lead to amputation, a two-pronged approach involving patients and the public at large may be more effective. Given the long-term effects of diabetes and the natural history of peripheral arterial disease progression, compounded by the high likelihood of complications in untreated patients, effort should be placed on improving public awareness of the underlying conditions and presenting symptoms. A study by Hirsch et al. was the first survey of public knowledge regarding lower extremity peripheral arterial disease [29]. A cross-sectional telephone survey of 2501 adults over 50 was performed to determine symptom knowledge, an awareness of risk factors, a perception of causes, and other factors. This study found that only 26% of adults surveyed were familiar with the term peripheral arterial disease (PAD); in contrast, 67.1% and 73.9% of the study group were familiar with the terms “stroke” and “coronary artery disease,” respectively [29]. Interestingly, rare conditions, such as multiple sclerosis and cystic fibrosis, had higher name recognition rates, highlighting the potential importance of public campaigns to spread awareness. These efforts can encourage patients who recognize potential peripheral arterial disease symptoms, such as claudication, to pursue regular healthcare, which will limit the risk of subsequent complications.

From a diabetic screening perspective, hemoglobin A1c (HbA1c) testing is a common screening measure employed to diagnose and longitudinally manage diabetics. Zhao et al. studied a prospective cohort of 19,808 African American and 15,560 Caucasian diabetics for an average of 6.83 years in order to determine the relative risk of lower extremity amputation. Importantly, they focused on patients from low-income communities, determining that a graded relationship between HbA1c existed with amputation among both groups. Furthermore, this relationship existed even after controlling for smoking status, blood pressure, and income, indicating the potential for HbA1c as a risk predictor for amputation [30]. Following this, a more recent study by Suckow et al. analyzed Medicare data on over 11 million diabetics between 2002 and 2012, following them for 6.6 years in order to determine the effects of regular screening on amputation risk. They determined that the use of HbA1c, regardless of frequency, decreased the amputation risk across all races studied by 15%. Frequent testing was associated with a 39% decrease in amputation risk [31].

Based on these studies, it is evident that patient education programs emphasizing the importance of peripheral arterial disease screening, especially in those with diabetes, have a positive impact on limb outcomes. However, individual patient education, as well as public awareness campaigns, need to be targeted towards the audience who is at highest risk. This ranges from national publicity campaigns that can increase the general awareness of the underlying conditions and potential outcomes, but also local community-organized events that connect care providers with patients and provide sufficient time for patient-provider interaction. These efforts are important, given that lower educational levels have been associated with higher rates of long-term mortality after amputation [32]. Specifically, five-year mortality for amputees was lower for high-school graduates when compared to those who had not finished the degree (62.6% versus 84.3%) [32-33]. Finally, while patient education may provide an impetus for individuals to seek preventative vascular care, there also exists a variation among practitioners regarding the diagnostic and treatment approach. For example, Medicare data for peripheral arterial disease patients as recent as 2008 demonstrated a variation in amputation rates based on geographic region. Specifically, they noted that areas with high amputation rates tended to cluster together, indicating a potential lack of access to health care within these areas [27]. Therefore, it is necessary to continue developing guidelines to help further standardize care protocols.

Effect of healthcare coverage and data collection

The previously noted interventions are beneficial, but it should be considered that not all patients have access to a provider or the financial means to obtain care commensurate with their current health status. Many amputees are older, due to ischemic complications secondary to peripheral arterial disease or diabetes requiring decades to manifest. Therefore, while many amputees may have Medicare coverage–with a smaller proportion being under the age limit for coverage–the preventative measures required to limit damage need to be started as early as possible. Considering that two patients with Medicare coverage can have significantly different rates of early life health screening and preventative efforts, the eventual outcomes for which data is collected varies vastly. One study using Kaiser Permanente data from 1995 to 1998 highlights the potential benefits of increased insurance coverage; note that Kaiser Permanente has a prepaid health plan that provides comprehensive health services to its 2.9 million members. Of these, 62,432 diabetics were followed and tracked based on the discharge diagnosis in order to estimate the risk for amputation. They concluded that there was no statistically significant difference between Caucasians and African Americans for lower extremity amputation, even after adjustment for age and sex [34]. Although this single study does not indicate the effects of broader coverage and its impact on different regions (with Kaiser Permanente covering areas in California, Colorado, District of Columbia, Georgia, Hawaii, Maryland, Oregon, Virginia, and Washington) or under different care systems, it does indicate the potential to mitigate racial differences in amputation by providing broader coverage.

A more recent study analyzed the effects of the 2006 expanded insurance coverage program in Massachusetts. This program expanded coverage to 98% of state residents, which highly benefited non-Caucasian residents [35]. The rates of uninsured Hispanics and African Americans decreased by 44% and 77%, respectively [35]. Using data from 2001 to 2009 from Massachusetts, New York, Maryland, and New Jersey, the study sought to determine potential changes in outcomes before and after the insurance expansion. They found that prior to 2006, non-white patients had a 12% higher probability of peripheral arterial disease, in both Massachusetts and control states. However, data collected after the expansion demonstrated no differences by race in Massachusetts while the control states maintained the disparity. Furthermore, the amputation risk difference between non-whites and whites decreased from a statistically significant 8.5% to a statistically insignificant 3.9% after the expansion. After the reform, there was no differential change in the mean cost of care between Massachusetts and the control states; however, the average length of stay decreased by 1.24 days in Massachusetts [35]. When accounting for the long-term costs of amputation, there may even be a cost-benefit with expanded coverage while improving quality of care. However, this study must be taken in the context of the overall economic climate in which they were conducted. The Massachusetts study occurred in the middle of the 2008 economic recession, which could have influenced the primary outcomes in either direction. Furthermore, even though the control states were close geographically, this does not necessarily account for state-by-state variation in healthcare ecosystems. Nevertheless, this study does indicate the potential positive effect of expanded healthcare coverage at a statewide level, though there are many competing factors.

Finally, when considering interventions based on population-based datasets, it is imperative that the data collection is standardized. Currently, the largest scale studies are from the Medicare or National Inpatient Sample (NIS) datasets. As noted earlier though, these datasets have many limitations, least of which is the variation in healthcare ecosystems at the local level. There are additionally significant differences between reported amputation rates due to differences in factors such as case identification criteria, whether or not the study focuses on peripheral arterial disease, diabetics, or both diseases and if the outcome of interest is amputation or other. Therefore, while racial disparities have been shown, an analysis at a state level, such as in the Massachusetts study, may shed light on geographic and healthcare coverage disparities that can be targeted.

## Conclusions

Disparities in healthcare processes and outcomes have been well documented in the United States. The current body of literature and data analyses show a clear association between race, coexisting diseases, and the risk of a negative limb outcome. More progress needs to be made towards improving health in communities by innovative interventions that address the behavioral, social and environmental determinants of health. Thus, collaborative approaches that involve strong partnerships between communities, stakeholders, and the healthcare system are needed for addressing contextual factors influencing health within a defined geographic, to move towards sustainable initiatives.

## References

[1] Ziegler-Graham K, MacKenzie EJ, Ephraim PL, Travison TG, Brookmeyer R (2008). Estimating the prevalence of limb loss in the United States: 2005 to 2050. Arch Phys Med Rehabil.

[2] Owings MF, Kozak LJ (1998). Ambulatory and inpatient procedures in the United States, 1996. Vital Health Stat 13.

[3] Lefebvre KM, Lavery LA (2011). Disparities in amputations in minorities. Clin Orthop Relat Res.

[4] Dillingham TR, Pezzin LE, Mackenzie EJ (2002). Racial differences in the incidence of limb loss secondary to peripheral vascular disease: a population-based study. Arch Phys Med Rehabil.

[5] Lefebvre KM, Metraux S (2009). Disparities in level of amputation among minorities: implications for improved preventative care. J Natl Med Assoc.

[6] (2018). Overview of race and hispanic origin: 2010. https://www.census.gov/prod/cen2010/briefs/c2010br-02.pdf.

[7] Olokoba AB, Obateru OA, Olokoba LB (2012). Type 2 diabetes mellitus: a review of current trends. Oman Med J.

[8] Young BA, Maynard C, Reiber G, Boyko EJ (2003). Effects of ethnicity and nephropathy on lower-extremity amputation risk among diabetic veterans. Diabetes Care.

[9] Norgren L, Hiatt WR, Dormandy JA, Nehler MR, Harris KA, Fowkes FG (2007). Inter-society consensus for the management of peripheral arterial disease (TASC II). J Vasc Surg.

[10] Franklin H, Rajan M, Tseng CL, Pogach L, Sinha A, Mph M (2014). Cost of lower-limb amputation in U.S. veterans with diabetes using health services data in fiscal years 2004 and 2010. J Rehabil Res Dev.

[11] MacKenzie EJ, Jones AS, Bosse MJ (2007). Health-care costs associated with amputation or reconstruction of a limb-threatening injury. J Bone Joint Surg Am.

[12] Sinha R, van den Heuvel WJ, Arokiasamy P (2017). Factors affecting quality of life in lower limb amputees. Prosthet Orthot Int.

[13] Akyol Y, Tander B, Goktepe AS, Safaz I, Kuru O, Tan AK (2013). Quality of life in patients with lower limb amputation: does it affect post-amputation pain, functional status, emotional status and perception of body image?. J Musculoskelet Pain.

[14] Jones WS, Patel MR, Dai D, Vemulapalli S, Subherwal S, Stafford J, Peterson ED (2013). High mortality risks after major lower extremity amputation in Medicare patients with peripheral artery disease. Am Heart J.

[15] Higgins TF, Klatt JB, Beals TC (2010). Lower extremity assessment project (LEAP) - the best available evidence on limb-threatening lower extremity trauma. Orthop Clin North Am.

[16] Dillingham TR, Pezzin LE, MacKenzie EJ (2002). Limb amputation and limb deficiency: epidemiology and recent trends in the United States. South Med J.

[17] Goldberg JB, Goodney PP, Cronenwett JL, Baker F (2012). The effect of risk and race on lower extremity amputations among Medicare diabetic patients. J Vasc Surg.

[18] Holman KH, Henke PK, Dimick JB, Birkmeyer JD (2011). Racial disparities in the use of revascularization before leg amputation in Medicare patients. J Vasc Surg.

[19] Baser O, Verpillat P, Gabriel S, Wang L (2013). Prevalence, incidence, and outcomes of critical limb ischemia in the US Medicare population. Vascular Disease Management.

[20] Hughes K, Seetahal S, Oyetunji T (2014). Racial/ethnic disparities in amputation and revascularization: a nationwide inpatient sample study. Vasc Endovascular Surg.

[21] (2018). Health insurance coverage in the United States: 2015. https://www.census.gov/content/dam/Census/library/publications/2016/demo/p60-257.pdf.

[22] Goodney PP, Holman K, Henke PK (2013). Regional intensity of vascular care and lower extremity amputation rates. J Vasc Surg.

[23] Humphries MD, Brunson A, Hedayati N, Romano P, Melnkow J (2016). Amputation risk in patients with diabetes mellitus and peripheral artery disease using statewide data. Ann Vasc Surg.

[24] Varma P, Stineman MG, Dillingham TR (2014). Epidemiology of limb loss. Phys Med Rehabil Clin N Am.

[25] Mustapha JA, Fisher BT, Sr. Sr., Rizzo JA (2017). Explaining racial disparities in amputation rates for the treatment of peripheral artery disease (PAD) using decomposition methods. J Racial Ethn Health Disparities.

[26] Newhall K, Spangler E, Dzebisashvili N, Goodman DC, Goodney P (2016). Amputation rates for patients with diabetes and peripheral arterial disease: the effects of race and region. Ann Vasc Surg.

[27] Jones WS, Patel MR, Dai D, Subherwal S, Stafford J, Calhoun S, Peterson ED (2012). Temporal trends and geographic variation of lower extremity amputation in patients with peripheral artery disease: results from U.S. Medicare 2000-2008. J Am Coll Cardiol.

[28] Amin L, Shah BR, Bierman AS, Lipscombe LL, Wu CF, Feig DS, Booth GL (2014). Gender differences in the impact of poverty on health: disparities in risk of diabetes-related amputation. Diabet Med.

[29] Hirsch AT, Murphy TP, Lovell MB (2007). Gaps in public knowledge of peripheral arterial disease: the first national PAD public awareness survey. Circulation.

[30] Zhao W, Katzmarzyk PT, Horswell R (2013). HbA1c and lower-extremity amputation risk in low-income patients with diabetes. Diabetes Care.

[31] Suckow BD, Newhall KA, Bekelis K (2016). Hemoglobin A1c testing and amputation rates in black, hispanic, and white Medicare patients. Ann Vasc Surg.

[32] Corey MR, Julien JS, Miller C (2012). Patient education level affects functionality and long term mortality after major lower extremity amputation. Am J Surg.

[33] Chin MH, Goddu AP, Ferguson MJ, Peek ME (2014). Expanding and sustaining integrated health care-community efforts to reduce diabetes disparities. Health Promot Pract.

[34] Karter AJ, Ferrara A, Liu JY, Moffet HH, Ackerson LM, Selby JV (2002). Ethnic disparities in diabetic complications in an insured population. JAMA.

[35] Loehrer AP, Hawkins AT, Auchincloss HG, Song Z, Hutter MM, Patel VI (2016). Impact of expanded insurance coverage on racial disparities in vascular disease: insights from Massachusetts. Ann Surg.

